# A primer on skeletal dysplasias

**DOI:** 10.1007/s11604-021-01206-5

**Published:** 2021-10-25

**Authors:** Atsuhiko Handa, Gen Nishimura, Malia Xin Zhan, D. Lee Bennett, Georges Y. El-Khoury

**Affiliations:** 1 Department of Radiology, University of Iowa Hospitals and Clinics, Iowa City, IA USA; 2 Department of Radiology, Boston Children’s Hospital and Harvard Medical School, 300 Longwood Ave, Boston, MA 02115 USA; 3 Center for Intractable Diseases, Saitama Medical University Hospital, Saitama, Japan; 4 University of Iowa Roy J. and Lucille A. Carver College of Medicine, Iowa City, IA USA

**Keywords:** Skeletal dysplasia, Aunt Minnie, Radiology board examination

## Abstract

Skeletal dysplasia encompasses a heterogeneous group of over 400 genetic disorders. They are individually rare, but collectively rather common with an approximate incidence of 1/5000. Thus, radiologists occasionally encounter skeletal dysplasias in their daily practices, and the topic is commonly brought up in radiology board examinations across the world. However, many radiologists and trainees struggle with this issue because of the lack of proper resources. The radiological diagnosis of skeletal dysplasias primarily rests on pattern recognition—a method that is often called the “Aunt Minnie” approach. Most skeletal dysplasias have an identifiable pattern of skeletal changes composed of unique findings and even pathognomonic findings. Thus, skeletal dysplasias are the best example to which the Aunt Minnie approach is readily applicable.

## Introduction

Skeletal dysplasias encompass a heterogeneous group of over 400 genetic disorders. They are individually rare, but collectively rather common, with an approximate incidence of 1/5000. Thus, radiologists occasionally encounter skeletal dysplasias in their daily practice, and the topic is commonly brought up on radiology board examinations across the world. However, many radiologists and trainees struggle with the issue because of the lack of proper resources.

In general, the radiological diagnosis rests on pattern recognition. This method is often called the “Aunt Minnie” approach—a term coined by Dr. Edward “Ed” Neuhauser (Former Chief of Radiology at Boston Children’s Hospital) and popularized by Dr. Benjamin “Ben” Felson (Former Director and a renowned chest radiologist at the University of Cincinnati). The Aunt Minnie approach has been explained by this analogy: if you knew your aunt named Minnie well, then you would easily recognize her in a crowd of people; however, recognizing her would be difficult for someone who had never seen your Aunt Minnie before. Most skeletal dysplasias have an identifiable pattern of skeletal changes composed of unique findings and even pathognomonic findings. Thus, skeletal dysplasias are one of the best examples to which the Aunt Minnie approach is readily applicable.

This article is mainly written for residents preparing for their board examinations and follows the official study guide for The American Board of Radiology Core Exam (https://www.theabr.org/). We will demonstrate the "Aunt Minnie" radiological findings and patterns of classic skeletal dysplasias and review their clinical and genetic features (genetic features summarized in Table 1). All the conditions that fall under the category of skeletal dysplasias in the study guide have been included in this review. Once readers finish this article, they should be able to recognize the characteristic imaging features of various skeletal dysplasias.

**Table 1:** Major skeletal dysplasias with causative genes

Achondroplasia and Thanatophoric dysplasia	Autosomal dominant. Gain-of-function mutation in *FGFR3.* Since over 99% of achondroplasia patients have the same mutation, the phenotype is extremely homogeneous
Chondrodysplasia punctata	Various
Jeune ATD (Skeletal ciliopathies)	Autosomal recessive. Many genes have been found
Multiple epiphyseal dysplasia	Autosomal dominant and recessive. Autosomal dominant MEDs are caused by mutations in *COMP* (MED1), *COL9A2* (MED2), *COL9A3* (MED3), *COL9A1* (MED6), and *MATN3* (MED5)
SEDC	Autosomal dominant. *COL2A1* mutations
Mucopolysaccharidosis	Autosomal recessive. Mutations depend on the subtype
Albright Hereditary Osteodystrophy	Autosomal dominant. PHP-1A and PPHP are caused by inactivation of the Gs-protein alpha subunit encoded by *GNAS1*. *GNAS1* is a heavily imprinted gene: mutations in the maternal-derived allele lead to PHP-1A, while those in the paternal-derived allele to PPHP
Osteogenesis imperfecta	Most cases are autosomal dominant and caused by heterozygous mutations in *COL1A1* or *COL1A2*
Hypophosphatasia	Autosomal recessive and dominant forms. Mutations of *TNSALP*
Osteopetrosis	Autosomal recessive (infantile and intermediate forms), and autosomal dominant (late-onset form)
Pyknodysostosis	Autosomal recessive. Mutations in *CTSK* encoding cathepsin-K
Osteopoikilosis	Autosomal dominant. *LEMD3* mutation
Osteopathia Striata	Mostly sporadic
Melorrheostosis	Mostly sporadic
Metaphyseal dysplasia (Pyle disease)	Autosomal recessive. *SFRP4* mutation
Diaphyseal dysplasia (Camurati–Engelmann disease)	Autosomal dominant and caused by domain-specific mutations in *TGBF1*
Pachydermoperiostosis (primary hypertrophic osteoarthropathy)	Various. Autosomal recessive form is caused by mutations in *SLCO2A1* or *HPGD*. The gene causing autosomal dominant form is unknown
Infantile cortical hyperostosis (Caffey disease)	Autosomal dominant. Mutation in *COL1A1* (a site-specific mutation)
Cleidocranial dysplasia	Autosomal dominant. Caused by *RUNX2* mutations or deletions
Nail patella syndrome	Autosomal dominant. Caused by *LMX1B* mutation
Hereditary multiple exostoses	Autosomal dominant. *EXT1* or *EXT2* mutation. Malignant degeneration is caused by mechanisms involving other genes
Dysplasia epiphysealis hemimelica (Trevor disease)	Unknown
Enchondromatosis	Mostly sporadic
Polyostotic Fibrous dysplasia (McCune–Albright syndrome, Mazabraud)	Somatic mutations in *GNAS1*

## Achondroplasia and Thanatophoric dysplasia

**Fig. 1:**
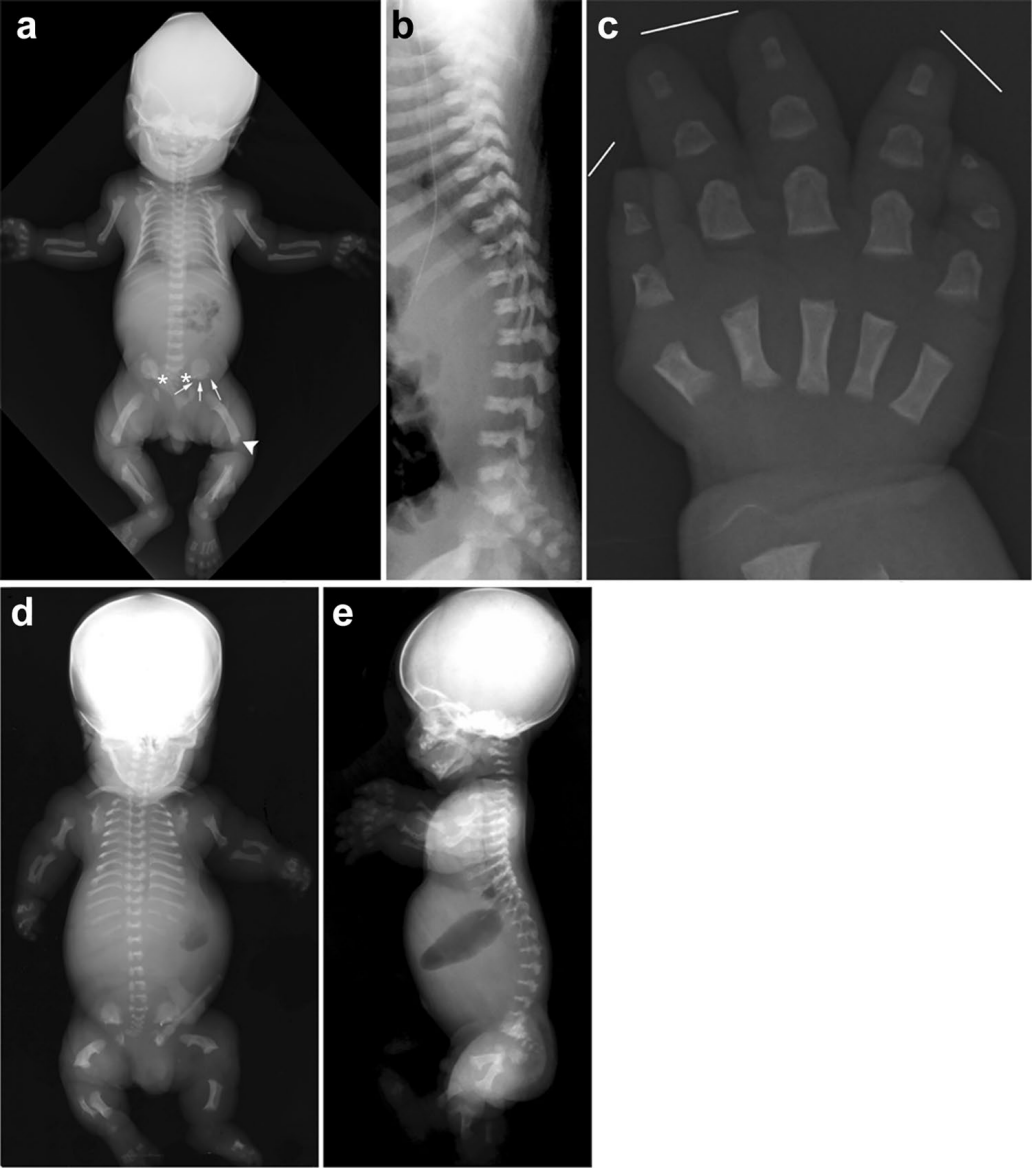
**a**, **b** A neonate with achondroplasia. **a** Radiographs show a disproportionately large skull, a narrow thorax, absence of normal increase in caudal interpedicle distances, small iliac bones with short sacrosciatic notches (asterisks) and trident-appearing acetabula (corniculate protrusion of the lateral, middle, and medial edge of the triradiate cartilage; arrows), ovoid lucency in the proximal femora, short and broad tubular bones associated with metaphyseal cupping, lateral slanting of the end of the distal femoral ends (arrowhead), short tibiae leading to relative elongation of the fibulae, and **b** “bullet-shaped” flat vertebral bodies and narrow spinal canal (on lateral view). **c** A frontal hand radiograph of another neonate with achondroplasia shows radial slanting of the 2nd metacarpal end and proximal phalangeal base, and a so-called “trident hand” (unable to oppose the 3rd and 4th fingers; three white lines). **d**, **e** A stillbirth with thanatophoric dysplasia. Radiographs show a disproportionately large skull, an extremely narrow thorax, severe platyspondyly (i.e., flat spine), caudally decreased interpedicle distances, small iliac bones with narrow sacrosciatic notches and trident-shaped acetabula, bowed femora (termed “French telephone receiver femora”), ovoid lucency of the proximal humeri/ femora (caused by metaphyseal cupping), metaphyseal cupping of the long bones. Note all the skeletal changes are qualitatively similar to, but quantitatively much more severe than those of achondroplasia

Achondroplasia is the most common short-limbed skeletal dysplasia [1]. This well-known skeletal dysplasia is caused by gain-of-function mutations in the *FGFR3* gene, which cause generalized impairment of endochondral bone formation but not intramembranous bone formation [2]. It is intriguing that almost all achondroplastic individuals have the same mutation, and the clinical manifestations are quite homogeneous among patients. The clinical features include short stature with rhizomelic (proximal segment) shortening of the limbs, inability to oppose the third and fourth fingers (“trident hand”), disproportionately large skull with prominent forehead and mid-face recession with relative prognathism (protrusion of the jaws), thoracolumbar kyphosis with prominent lumbosacral lordosis in childhood, and rapidly progressive stenosis of the foramen magnum after birth that may lead to hydrocephalus, apnea, quadriplegia, and even sudden death in infancy and early childhood. The key radiological findings include a combination of impaired endochondral bone formation and preserved intramembranous ossification. In tubular bones, for example, abnormal longitudinal bone growth and unaffected transverse bone growth lead to short and relatively broad bones with metaphyseal cupping (Fig. 1a–c). Proximal femoral radiolucency seen in neonates and infants is pathognomonic.

Thanatophoric dysplasia is the severe variant of achondroplasia [3]. It is the most common lethal skeletal dysplasia, caused by different *FGFR3* mutations that give rise to more severe biological consequences than seen in achondroplasia. The term “thanatophoric” derives from a Greek word *thanatophorus* meaning death bearing [4]. The clinical features include a narrow thorax leading to respiratory distress and extremely short limbs. Type 1 thanatophoric dysplasia shows anterolateral bowing of the upper leg and posteromedial bowing of the lower leg. The key radiological pattern is essentially the same with achondroplasia but much more striking (Fig. 1d, e). Notably, a combination of femoral bowing and proximal femoral radiolucency gives a distinctive appearance termed “French telephone receiver femora”.

## Chondrodysplasia punctata

**Fig. 2:**
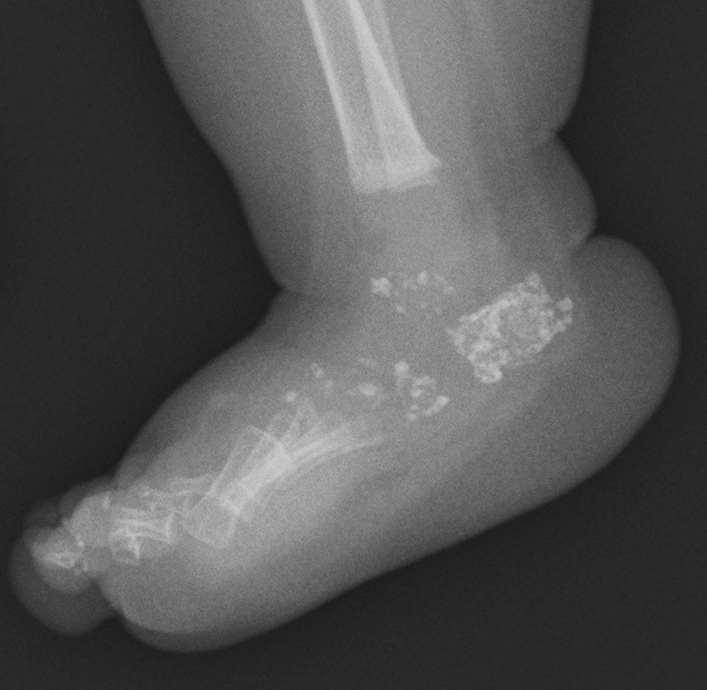
An infant with chondrodysplasia punctata. Multiple puncta are seen in the tarsal bones and the phalanges

Chondrodysplasia punctate (CDP) is a heterogeneous group of disorders characterized by “puncta” or “stippled epiphyses” that refer to punctate calcifications in the cartilage, particularly prominent in the epiphyseal cartilage [5]. CDP is divided into a few subgroups that have different clinical manifestations and different genetic causes, including Conradi–Hunermann type, rhizomelic type, brachytelephalangic type [6]. Each type has a unique pattern of puncta along with distinctive skeletal and extra-skeletal changes. However, it is intriguing that all types share severe nasomaxillary hypoplasia termed “Binder face”. Stippled epiphyses are seen from birth to 2–4 years of life (Fig. 2) which then evolve into epiphyseal dysplasia (irregular epiphyseal ossification). Puncta may also occur in acquired disorders. Examples include warfarin embryopathy and maternal collagen vascular diseases (e.g., systemic lupus erythematosus) that commonly masquerade as CDP brachytelephalangic type.

## Jeune asphyxiating thoracic dysplasia

**Fig. 3:**
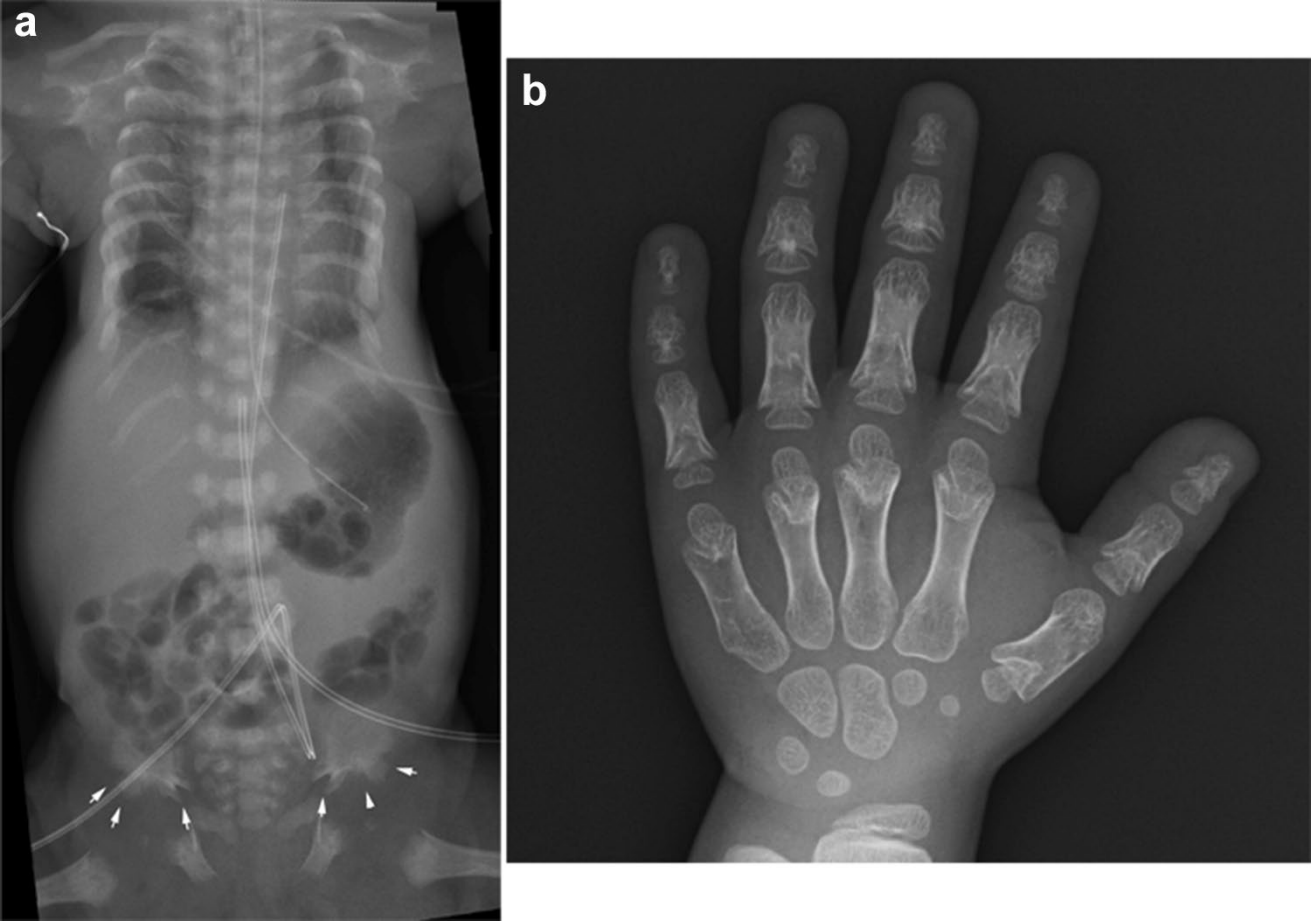
**a** A neonate with Jeune asphyxiating thoracic dysplasia. Radiograph shows markedly short ribs, and trident-shaped ilia (arrows). Severe brachydactyly without polydactyly were also noted (not shown). Premature ossification of the proximal femoral epiphyses is distinctive. As opposed to achondroplasia, there is no caudally decreased interpedicle distance or ovoid lucency of the proximal femora. (Reprinted from Handa, A, et al. Jpn J Radiol. 2020;38(3):193–206). **b** A child with the same diagnosis. Radiograph shows cone-shaped epiphyses involving both the phalanges and the metacarpals. Cone-shaped phalangeal epiphyses and inverted tear-shaped metacarpal epiphyses invaginate into cupped metaphyses. Premature fusion of the growth plate is also seen in the middle and distal phalanges. (Reprinted from Handa, A, et al. Jpn J Radiol. 2020;38(3):193–206)

Jeune asphyxiating thoracic dysplasia (ATD) is the most common condition among “skeletal ciliopathy”—a group of skeletal disorders caused by abnormal primary cilia [7]. The clinical features of ATD include thoracic hypoplasia with respiratory distress, short extremities, and severe brachydactyly. The extra-skeletal complications include progressive renal disease evolving into end-stage renal disease during childhood, occasionally associated with liver fibrosis. The key radiological findings include short ribs, short ilia with “trident”-appearing ilia, shortened long tubular bones, brachydactyly with “cone”-shaped epiphyses rarely accompanied by postaxial polydactyly, and a normal skull and spine (Fig. 3). Premature ossification of the proximal femora is a unique finding.

## Multiple epiphyseal dysplasia

**Fig. 4:**
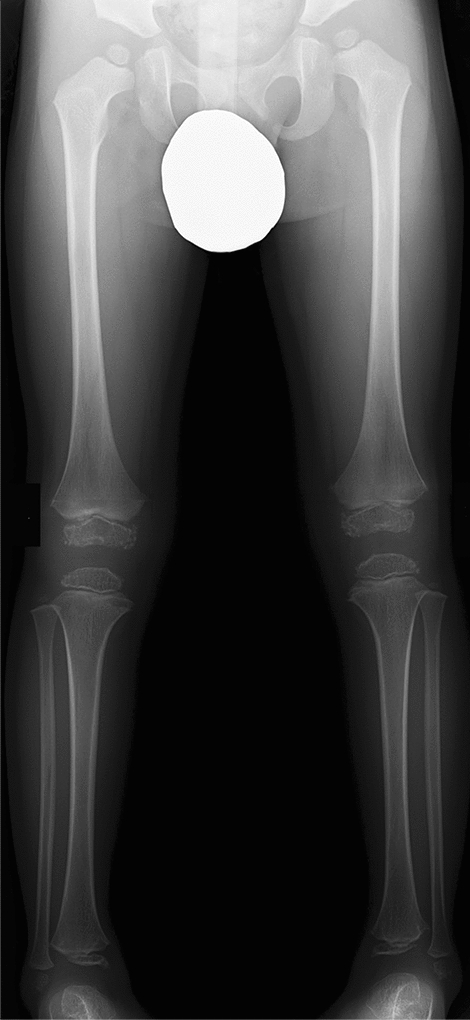
A 5-year-old child with multiple epiphyseal dysplasia, autosomal dominant type. Radiographs show delayed, disorganized epiphyseal ossification including small and round capital femoral epiphyses and irregular epiphyseal ossification of the knee

Multiple epiphyseal dysplasia (MED) comprises a diverse group of disorders characterized by abnormal epiphyseal ossification [8]. It has been known that several genes are responsible for MED [9]. The most common type is caused by *COMP* mutations. The clinical features of MED include postnatal short stature as well as joint pain and joint laxity. Affected individuals are normal at birth and during infancy but then develop mild to moderate short stature that begins in early childhood and progresses over childhood. The key radiological findings are, as is indicated by the disease name, delayed and irregular ossification of all epiphyses (Fig. 4), which later develops into premature degenerative joint disease. Although epiphyseal changes are the cardinal feature, mild metaphyseal and spinal dysplasia are rather common, especially those caused by *COMP* mutations.

## Spondyloepiphyseal dysplasia congenita

**Fig. 5:**
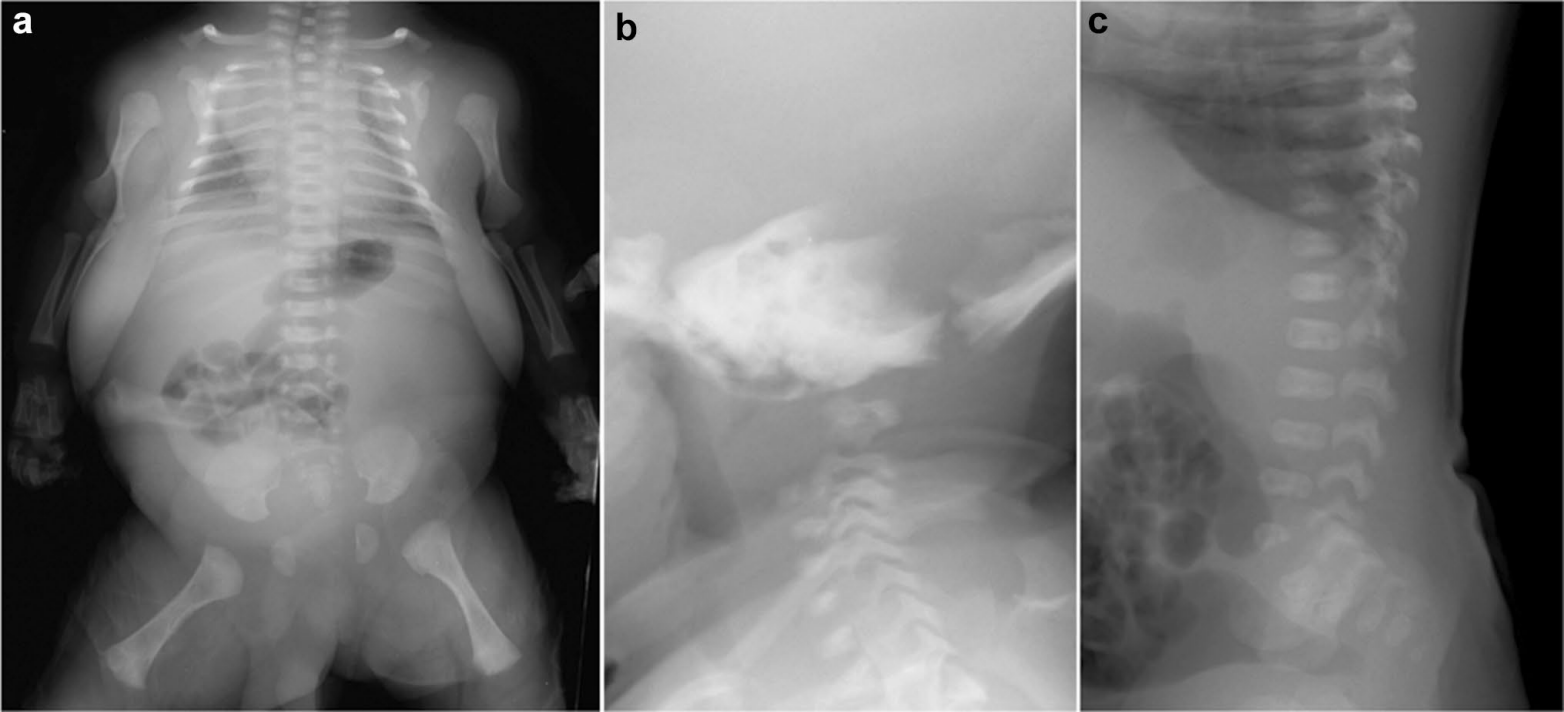
**a**–**c** A neonate with SEDC. Radiographs show a broad thorax and modest platyspondyly (flattening of the vertebral bodies) due to delayed vertebral ossification. The delayed ossification is prominent in the dorsal vertebral bodies, giving rise to pear-shaped vertebral bodies seen on the lateral view. The delayed ossification is more severe in the cervical spine and sacrum than in the thoracolumbar spine, creating anisospondyly (increased variability of the size of the vertebral bodies). The ilia are craniocaudally short, the pubic bones are absent, epiphyseal ossification of the distal femora is retarded (typically seen by 39 weeks of gestation), and the long bones show mild metaphyseal widening

Spondyloepiphyseal dysplasia congenita (SEDC) is the most common short-trunked bone dysplasia with predominant involvement of the spine and the epiphyses [10, 11]. SEDC is the common condition among type II collagenopathies—a group of disorders caused by abnormal type II collagen [12]. A clinical feature includes short stature varying in severity, predominantly caused by a short trunk and short neck. The proximal and middle segments of the limbs are modestly shortened, but the hands and feet are normal. Mid-face hypoplasia with cleft palate is another common finding. Type II collagen is an essential component of the eyes and inner ears as well as cartilage; thus, affected individuals often exhibit severe myopia, retinal detachment, and hearing problems. The key radiological finding can be summarized as delayed ossification of the juxta-truncal bones (i.e., spine, pelvis, and proximal epiphyses of long bones) (Fig. 5). Spinal ossification is most delayed in the cervical and sacral spine, and the shape of the thoracolumbar spine on the lateral view is often referred to as “pear-shaped” because of posterior constriction. A calvarial ossification defect dorsal to the foramen magnum is characteristic. Another diagnostic hallmark is delayed ossification of the pubic bones. Pubic bone ossification normally occurs in the early stage of the third trimester of the pregnancy, while the pubic bones are not ossified at birth in SEDC.

## Mucopolysaccharidosis (Dysostosis multiplex)

**Fig. 6:**
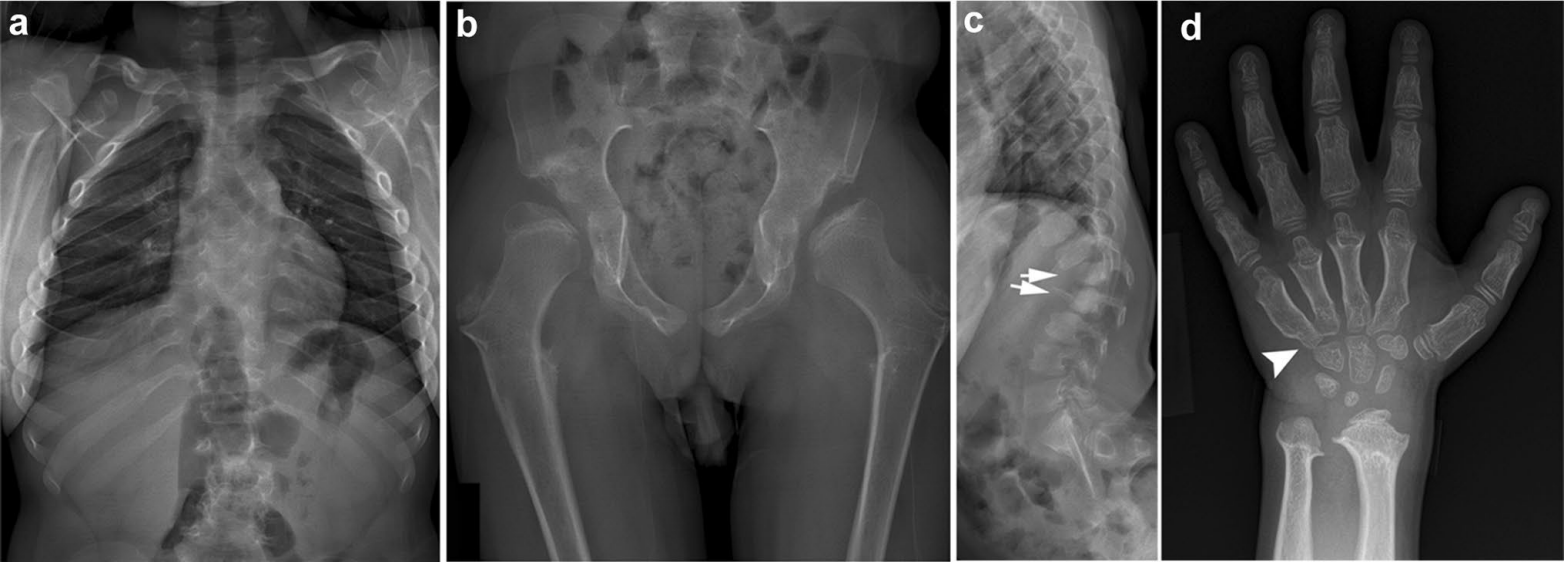
**a**–**d** An 11-year-old boy with Hurler syndrome. Radiographs show findings of dysostosis multiplex including **a** thick clavicles, paddle-shaped (or oar-shaped) broad ribs, **b** comma-shaped ilia, diaphyseal broadening and metaphyseal constriction of the long bones, **c** hook-shaped vertebral bodies (arrows) with posterior scalloping, **d** proximal pointing of the metacarpals (arrowhead), and bullet-shaped phalanges

Dysostosis multiplex is the term that refers to the skeletal phenotype seen in mucopolysaccharidoses, lysosomal storage diseases with absent or malfunctioning enzymes required for the breakdown of glycosaminoglycans (e.g., Hurler disease, Hunter disease, etc.) [13]. Regardless of the subtype, all mucopolysaccharidoses exhibit dysostosis multiplex of varying severity. The clinical features include characteristic coarse facial features, corneal clouding, short stature, and hepatosplenomegaly. The key radiological findings of dysostosis multiplex include a large and thick skull, J-shaped sella, thick clavicles, paddle-shaped (or oar-shaped) ribs, hook-shaped vertebral bodies with posterior scalloping, comma-shaped ilia, diaphyseal broadening and metaphyseal constriction of the long bones, proximal pointing of the metacarpals, and bullet-shaped phalanges (Fig. 6).

## Albright hereditary osteodystrophy

Albright hereditary osteodystrophy refers to a clinical manifestation of two disorders: pseudohypoparathyroidism (PHP) type IA and pseudo-pseudohypoparathyroidism (PPHP) [14, 15]. PHP and PPHP comprise a group of disorders that arise from abnormalities in the *GNAS* gene [16]. The phenotypic differences are related to genetic imprinting, i.e., maternal versus paternal transmission of the abnormal gene causes different clinical consequences. PHP is a group of endocrine disorders with end-organ hormone resistance to parathyroid hormone which manifests with hypocalcemia causing neurologic symptoms (e.g., seizures and tetany), hyperphosphatemia, and elevated parathyroid hormone. PHP is subclassified into types Ia, Ib, Ic, and II, which share parathormone unresponsiveness but has variable clinical and endocrine differences. PHP IA is the prototype of the group with classic endocrine abnormalities as well as Albright hereditary osteodystrophy. PPHP has Albright hereditary osteodystrophy but not parathormone unresponsiveness.

**Fig. 7:**
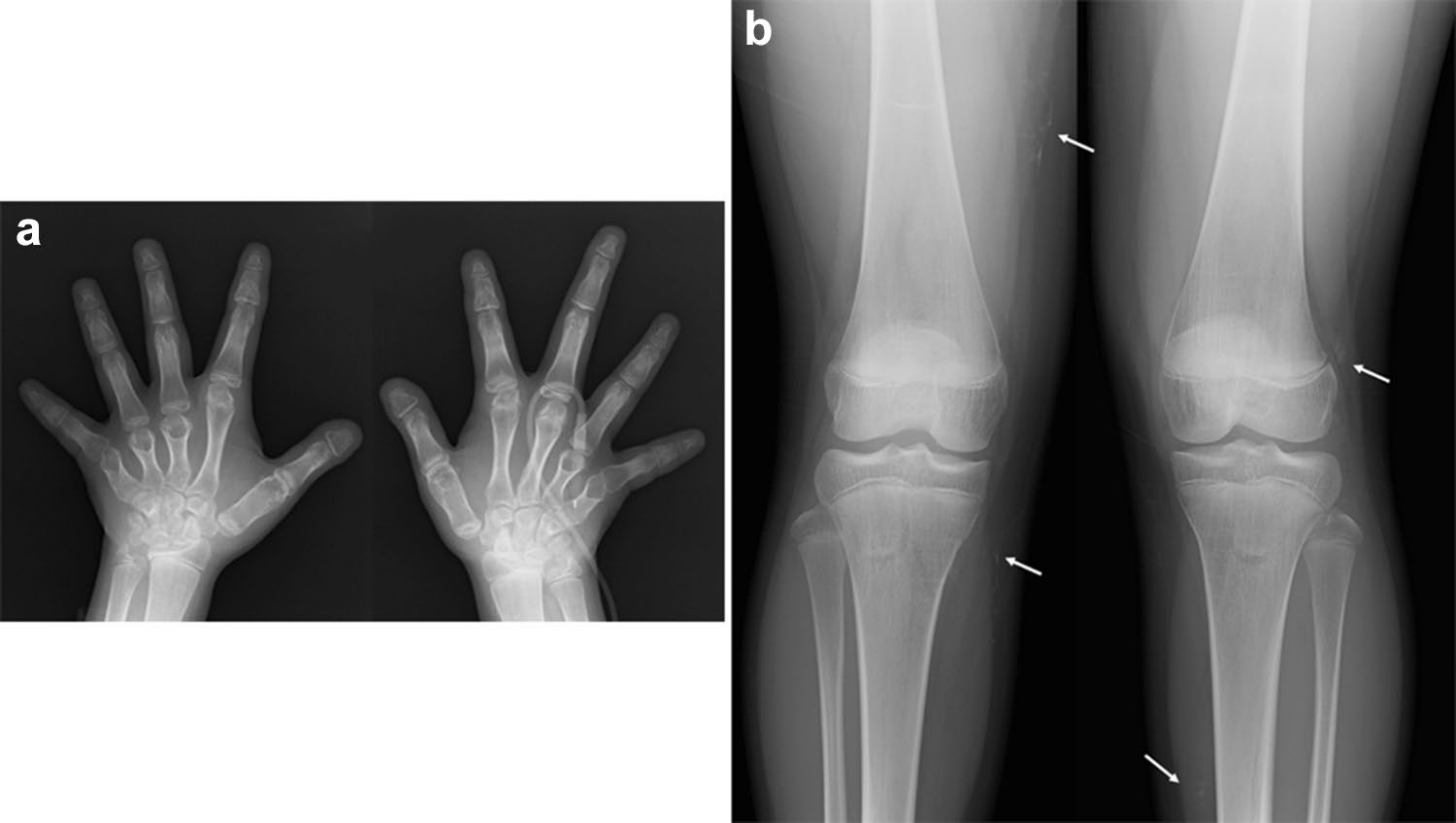
**a**, **b** A 12-year-old girl with Albright hereditary osteodystrophy. Radiographs show shortening of the ulnar metacarpals (type E brachydactyly) and short distal phalanges, particularly of the thumb, and periarticular soft tissue calcifications around both knees (arrows)

The clinical features of Albright hereditary osteodystrophy include short stature with brachydactyly, round face, obesity, and mild intellectual disability. The key radiological findings include type E brachydactyly (i.e., shortening of the metacarpals, most prominent in the 4th and 5th digits) and periarticular soft tissue calcifications (Fig. 7). Brain calcifications, particularly of the basal ganglia, are common.

## Osteogenesis imperfecta

**Fig. 8:**
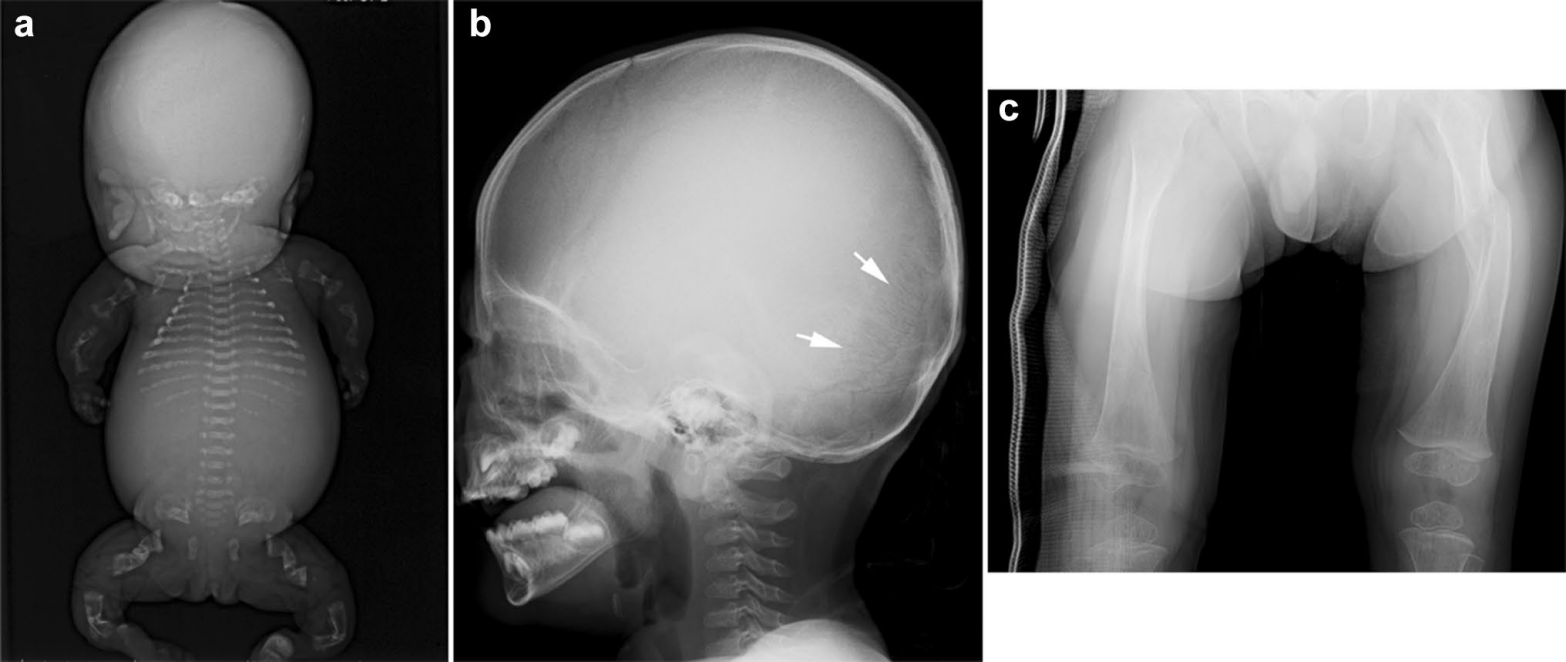
**a** A stillborn with osteogenesis imperfecta. Radiograph shows a beaded appearance of the ribs and an “accordion-like” wavy appearance of the long bones as a consequence of in utero multiple fractures. **b**, **c** A 2-year-old patient with osteogenesis imperfecta. Radiographs show multiple Wormian bones (arrow) due to defective calvarial ossification and generalized osteoporosis with a healing fracture of the right femoral shaft and a healed fracture of the left femoral shaft

Osteogenesis imperfecta is a group of genetic osteoporosis syndromes characterized by impaired intramembranous ossification caused by abnormal synthesis of type I collagen [17, 18]. The clinical features include bone fragility, joint laxity, soft tissue fragility, and blue sclerae due to transparency of the sclerae. Sillence’s classification (Table 2) has been used to categorize osteogenesis imperfecta into four subtypes [19]. Severe forms of osteogenesis imperfecta manifest in utero with multiple fractures and deformity/bowing of the limbs, whereas mild forms present with recurrent fractures after minor trauma. The key radiological findings include generalized osteoporosis and multiple fractures (Fig. 8). Defective calvarial ossification manifests with multiple Wormian bones and widely opened fontanels.

**Table 2:** Sillence Classification of osteogenesis imperfecta based on phenotypes (in Arabic numerals) and molecular basis (in Roman numerals), and related disorders

Types	Severity with phenotype	OI types (in Roman numerals) and disorders
1	Mild, non-deforming, with blue sclerae	I
2	Severe, perinatal lethal	II
3	Moderate to severe, progressively deforming	III, VI, VIII, IX, X
4	Moderate with wide variety	IV, IV, VII, XI, XII, XIII
5	Moderate with hyperplastic callus	V,
		Others:
		Osteoporosis-pseudoglioma syndrome, etc.
		Idiopathic juvenile osteoporosis,
		Bruck syndrome type 1 and type 2

The differential diagnosis between non-accidental injury and mild osteogenesis imperfecta can be challenging. The findings that point to the diagnosis of non-accidental injury include classic metaphyseal lesions, complex skull fractures, and posterior rib fractures, while findings in favor of osteogenesis imperfecta include multiple Wormian bones, skull deformities, and osteoporosis.

## Hypophosphatasia

**Fig. 9:**
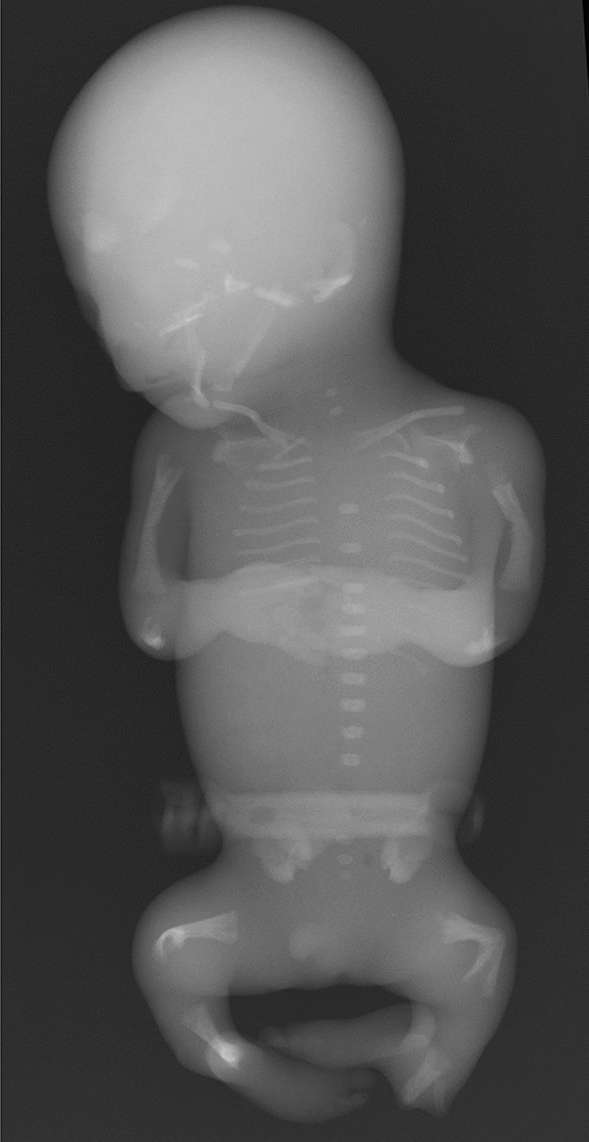
A stillborn with hypophosphatasia. Radiograph shows near-absent ossification of the skull (yet the frontal bones are preserved), short and thin ribs, small scapulae, absent ossification of the pubic and ischial bones, and sharp angulation of the long bones with metaphyseal defects extending into the diaphysis. Vertebral ossification is unequal. Some vertebral bodies are completely unossified, while others are well-ossified. The neural arches are thoroughly missing

Hypophosphatasia is characterized by impaired mineralization of the bones due to abnormal tissue-nonspecific alkaline phosphatase (ALP) [20, 21]. Hypophosphatasia comprises a wide continuous spectrum of severity, ranging from the most severe, perinatal lethal form to the mild adult form with only defective dental mineralization (odontohypophosphatasia) [22]. The clinical features correspond to disease severities. The perinatal lethal form presents with a soft head, deformed extremities, and thoracic hypoplasia with respiratory distress, as well as extremely low ALP levels. It most often manifests radiologically as near-total absence of ossification of the bones (“boneless fetus”) with preserved frontal bones (Fig. 9). The milder forms show variable degrees of rickets or osteomalacia and muscular hypotonia. However, the infantile and childhood forms usually show tongue-like radiolucencies extending from the metaphyses deeply toward the diaphyses, which differ from more uniformly distributed metaphyseal cupping and fraying, i.e., classical radiological signs of rickets. Recently, bone-targeted enzyme replacement therapy has been introduced to improve ossification and survival. Compared to osteogenesis imperfecta, patients with hypophosphatasia do not have Wormian bones.

## Osteopetrosis

**Fig. 10:**
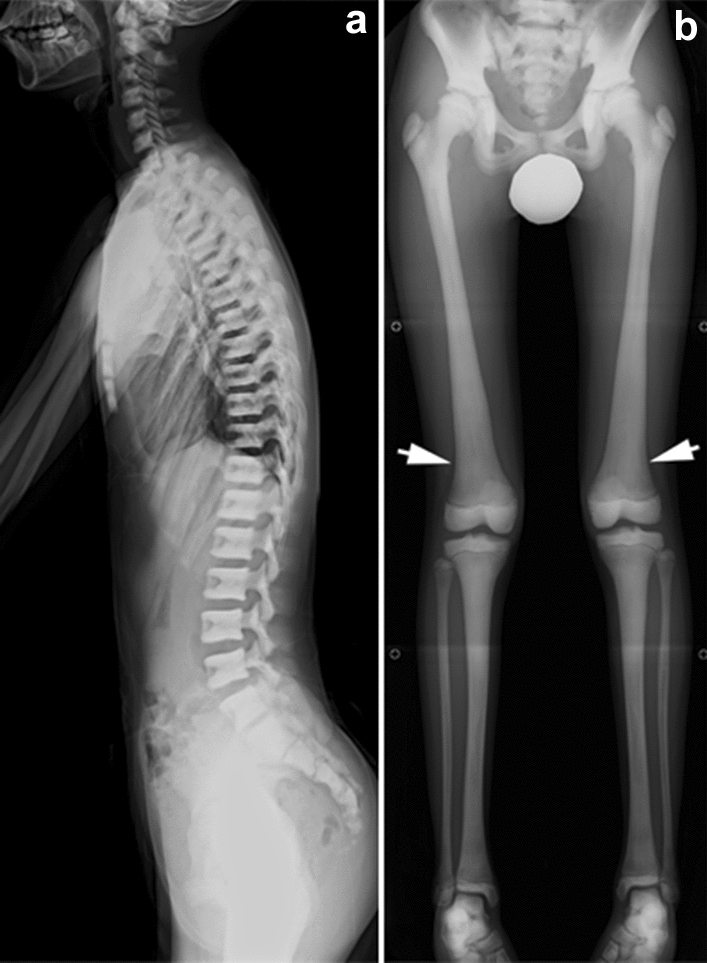
**a**, **b** A 14-year-old boy with adult type osteopetrosis. Radiographs show generalized increased bone density with poor corticomedullary differentiation and mild Erlenmeyer flask deformity of the long bones (arrows). Lateral view of the spine shows prominent superior and inferior endplate sclerosis, giving rise to a sandwich appearance of the vertebral bodies

Osteopetrosis is a heterogeneous group of conditions characterized by generalized bone sclerosis, caused by osteoclast dysfunction and impaired absorption of immature bone [23, 24]. Abundance of immature bone results in dense but brittle bones with obliterated bone marrow. Osteopetrosis can be classified into three groups based on the timing of onset: infantile, intermediate, and late-onset forms. The clinical features are: (1) infantile osteopetrosis manifesting with failure to thrive, bone marrow failure with hepatosplenomegaly and recurrent infection, and compressive neuropathy of the cranial nerves; (2) intermediate osteopetrosis presenting in early childhood with bone fragility, compression neuropathy, and occasionally mild bone marrow failure. (3) Late-onset osteopetrosis manifesting in late childhood to adolescence with susceptibility to fractures and dental caries. The key radiological findings include generalized increased bone density, occasionally associated with poor corticomedullary differentiation and indiscernible bone trabecular pattern, particularly in the infantile form (Fig. 10) [25]. A characteristic “bone-in-bone” appearance and sclerotic end plates causing “sandwich” vertebrae occur as a result of the cyclic nature of impaired immature bone resorption. Infantile and intermediate forms are associated with modeling failure causing the so-called “Erlenmeyer flask” deformity.

## Pyknodysostosis

**Fig. 11:**
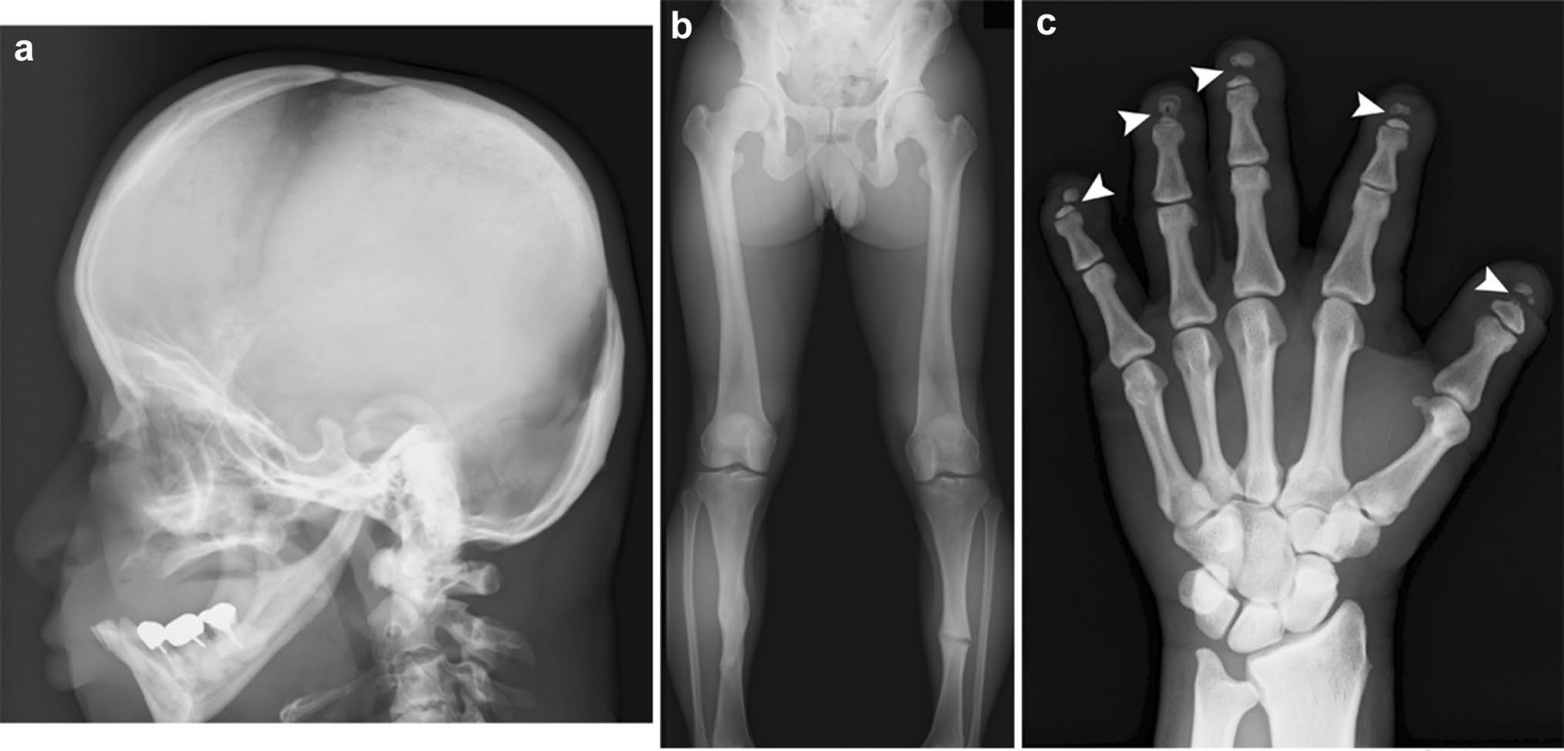
**a**–**c** A young adult with pyknodysostosis. Radiographs show craniofacial sclerosis with widely opened fontanels, severe micrognathia with an obtuse mandibular angle, and edentulous state due to repeated dental infections as well as generalized increased bone density with a preserved corticomedullary differentiation and trabecular pattern, healed fractures of both tibiae and **c** severe acroosteolysis (arrowheads)

Pyknodysostosis is a sclerotic bone disorder due to impaired absorption of immature bone accompanied by acroosteolysis (resorption of distal phalanges) and impaired calvarial ossification [26, 27]. The disorder is regarded as a special form of osteopetrosis. The clinical features include moderately short stature, bone fragility, susceptibility to dental infections and osteomyelitis, as well as short terminal phalanges. The key radiological findings include generalized bone sclerosis, mild diaphyseal constriction, and osteolysis of the distal phalanges [28]. Corticomedullary differentiation and bone trabecular pattern are discernible in pyknodysostosis (Fig. 11). Defective calvarial ossification results in wide fontanels and multiple Wormian bones. An obtuse mandibular angle is another hallmark of this disorder.

## Sclerotic skeletal dysplasias

**Fig. 12:**
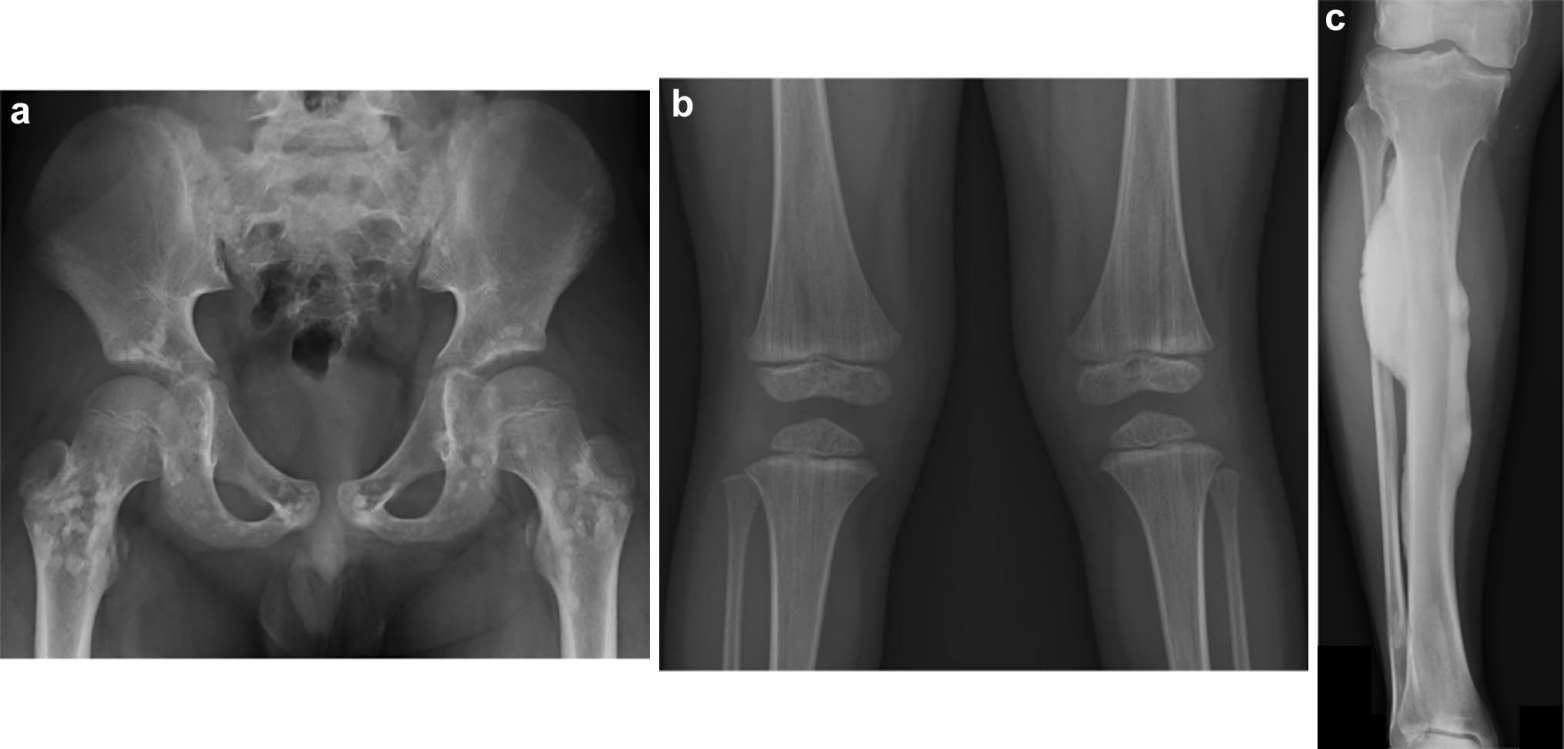
**a** A 10-year-old boy with osteopoikilosis. Radiograph shows small foci of bone sclerosis in different sizes and shapes (round, oval, lenticular) located in the pelvis and bilateral proximal femora. **b** A 4-year-old girl with osteopathia striata. Radiograph shows longitudinal striations in the distal femoral and proximal tibial metaphyses. **c** A 75-year-old male with melorheostosis. Radiograph shows dripping candle wax-like, eccentric hyperostoses distributed along the tibia

- Osteopoikilosis
  Osteopoikilosis is a benign condition characterized by multiple small sclerotic foci localized at the epi-metaphyses of the long bones [29]. The spherical to oblong sclerotic foci are aligned parallel to adjoining bone trabeculae (Fig. 12a). Affected individuals are usually asymptomatic and the bone lesions are usually found incidentally. Histopathologic findings are identical with those of benign bone islands.
- Osteopathia striata
  Osteopathia striata refers to longitudinal sclerotic striations in the metaphyses of the long bones (Fig. 12b) [30]. Osteopathia striata may be seen as an asymptomatic benign condition. Osteopathia striata associated with cranial sclerosis indicates an X-linked dominant disorder with male lethality, termed osteopathia striata–cranial sclerosis (OS–CS) [31, 32]. Affected females manifest with macrocephaly, cleft palate, and mild intellectual disability. Osteopathia striata may be associated with Goltz syndrome, alternatively termed focal dermal hypoplasia.
- Melorrheostosis
  Melorrheostosis is a condition characterized by distinctive cortical hyperostosis resembling “dripping candle wax” and named after the appearance (*melos*: limb; *rhein*: to flow; *ostos*: bone) in Greek (Fig. 12c) [33, 34]. The hyperostotic process causes pain and stiffness in the affected limbs. The other features include limb-length discrepancy, joint contracture, muscular atrophy, and erythematous skin changes. The dripping candle wax-like cortical hyperostosis may affect a single bone, but more commonly involves multiple continuous bones. The distribution tends to be unilateral and follows the sclerotomes (the areas of bones innervated by the same spinal segments). It should be noted that osteopoikilosis, osteopathia striata, and melorheostosis may concurrently occur. Such a case is termed mixed sclerosing bone dysplasia.

## Metaphyseal dysplasia (Pyle disease)

**Fig. 13:**
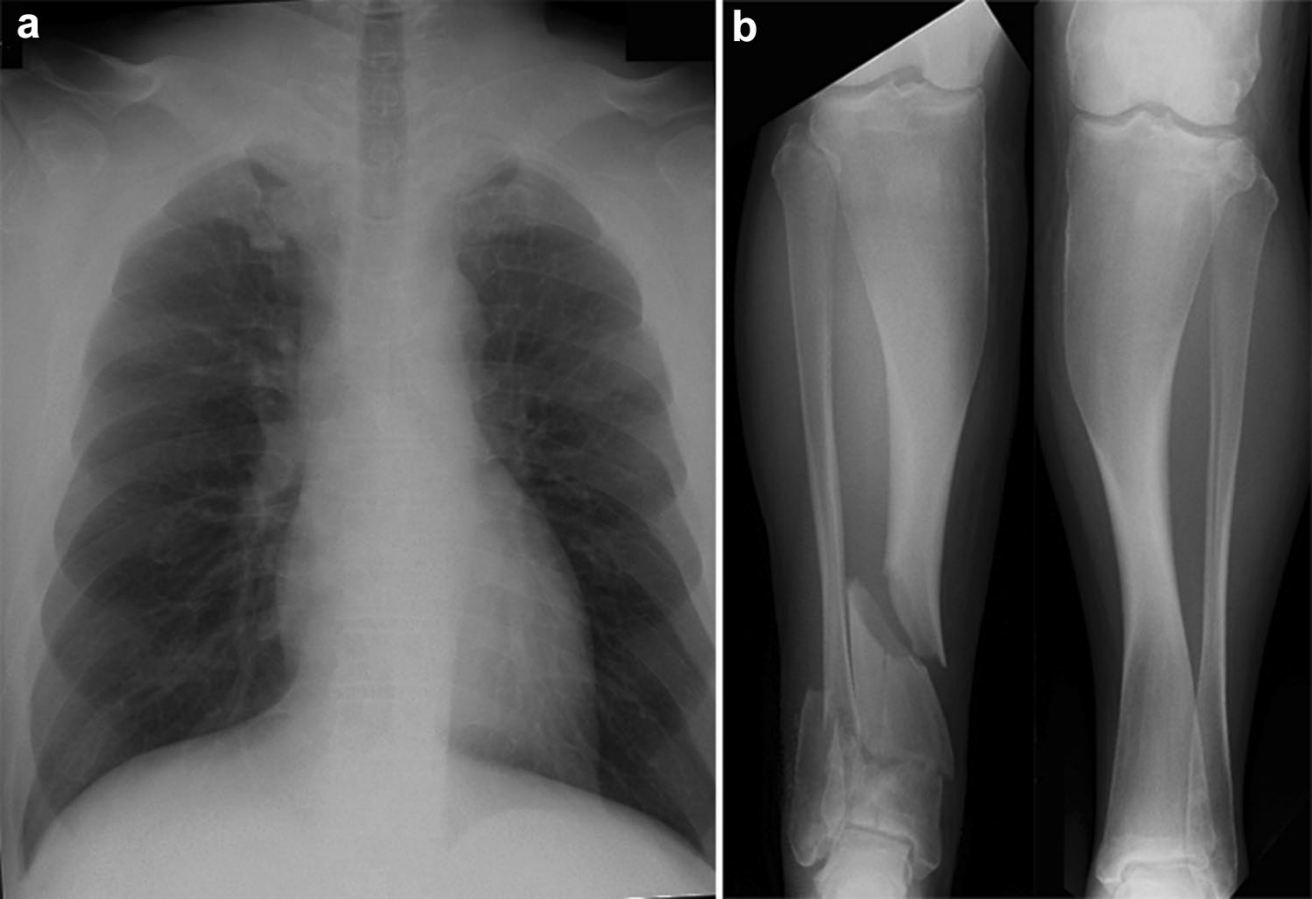
**a**, **b** An adult patient with metaphyseal dysplasia (Pyle disease). Radiographs show broad clavicles and ribs as well as significant expansion and thin cortices of the metaphysis and metadiaphyseal junction of the long bones giving rise to “Erlenmeyer flask” deformities. There are fractures of the right distal tibia and fibula

Metaphyseal dysplasia (Pyle disease) is characterized by striking metaphyseal broadening of the tubular bones [35, 36]. The clinical features include knock-knee or genu valgum. Most affected individuals are otherwise healthy, but a minority of patients have bone fragility. The key radiological finding is massive metaphyseal expansion extending into meta-diaphyseal junction (“Erlenmeyer flask” deformity), particularly prominent at the knee (Fig. 13). The cortices of the expanded meta-diaphyseal segments are very thin.

## Diaphyseal dysplasia (Camurati–Engelmann disease)

**Fig. 14:**
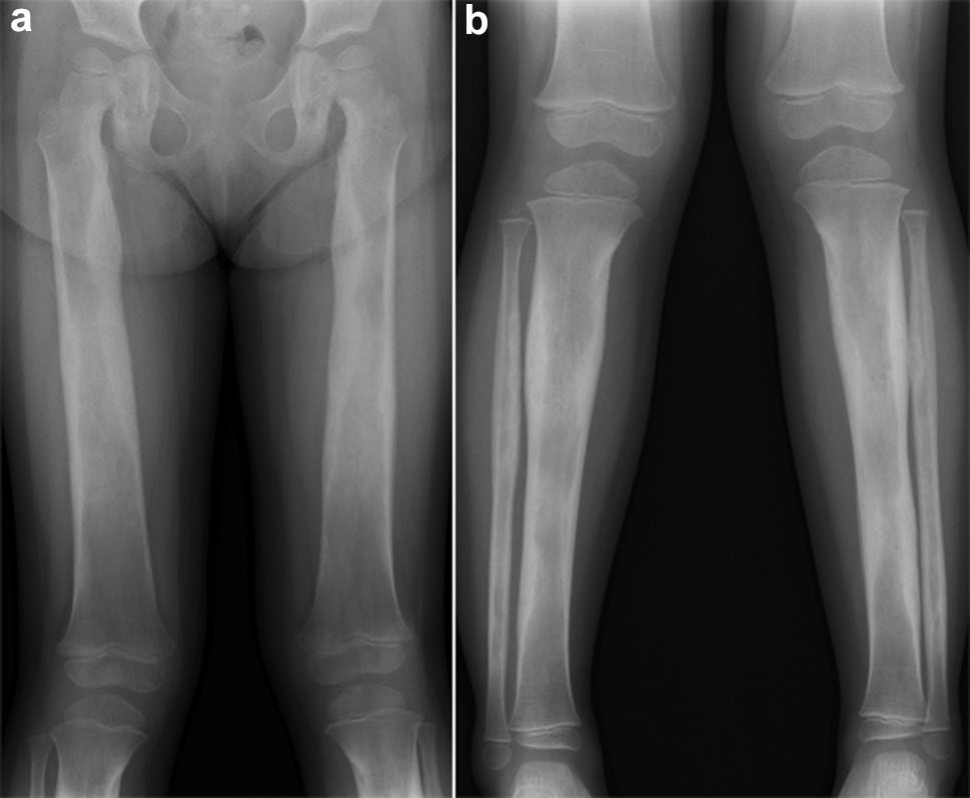
**a**, **b** A 3-year-old girl with diaphyseal dysplasia (Camurati–Engelmann disease). Radiograph shows cortical thickening with coinciding medullary narrowing of the long bones

Diaphyseal dysplasia (Camurati–Engelmann disease) is a sclerotic bone dysplasia characterized by irregular hyperostosis of the diaphyses [37–39]. The clinical features include leg pain, muscular weakness, and joint contracture. The key radiological finding is irregular cortical hyperostosis with narrowing of the medullary spaces of the diaphyses (Fig. 14). The metaphyses and epiphyses are not affected. The short tubular bones are typically not affected either. The flat bones show sclerosis of their diaphyseal equivalent parts. Skull base sclerosis can also be seen.

## Pachydermoperiostosis (primary hypertrophic osteoarthropathy)

**Fig. 15:**
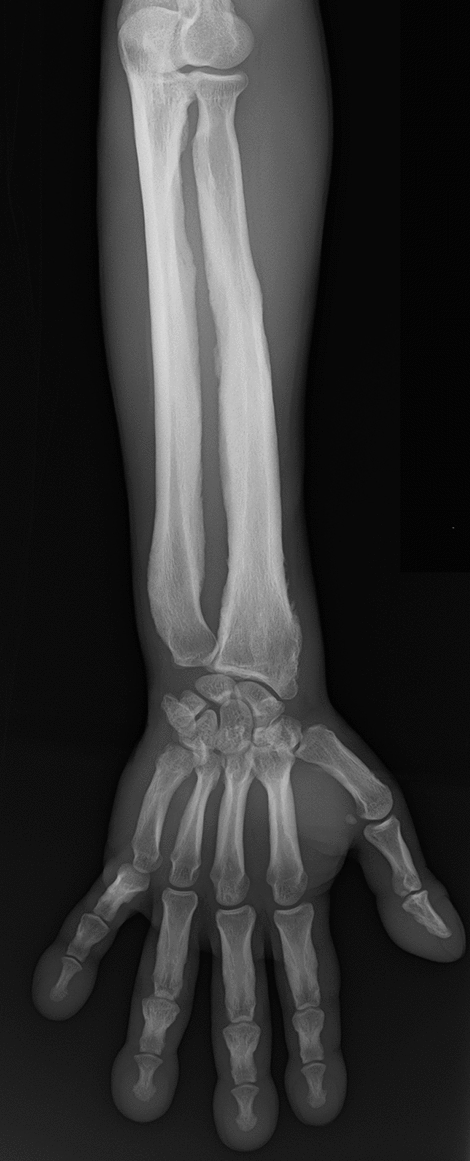
A 26-year-old male with pachydermoperiostosis. Radiograph shows irregular periosteal reaction of the short tubular bones and the long bones. Digital clubbing is also seen

Pachydermoperiostosis (primary hypertrophic osteoarthropathy) is a multi-system disorder involving the skin, soft tissue, and bone [40–42]. The clinical features include progressive dermal thickening (“pachydermia”) and digital clubbing which commonly develop during puberty. The key radiological findings include symmetric subperiosteal new bone formation in the long and short tubular bones and later diaphyseal expansion with reduced density and coarse trabeculation (Fig. 15). The latter finding contrasts with the narrowed medullary space seen in diaphyseal dysplasia.

Secondary hypertrophic osteoarthropathy that occurs as a result of a paraneoplastic syndrome, lung disease, or liver dysfunction shows similar radiographic findings to pachydermoperiostosis. Hypertrophy of the distal phalangeal tufts is more severe in secondary hypertrophic osteoarthropathy.

## Infantile cortical hyperostosis (Caffey disease)

**Fig. 16:**
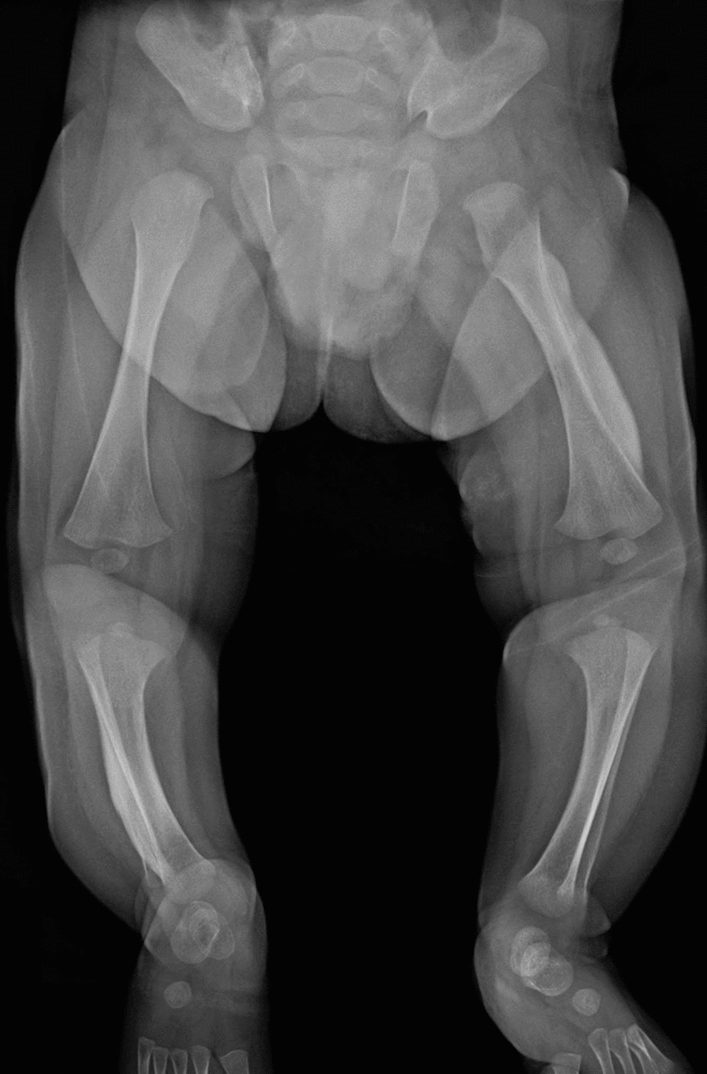
A 2-month-old infant with infantile cortical hyperostosis (Caffey disease). Radiograph shows cortical hyperostosis in the left femur and right tibia

Infantile cortical hyperostosis, alternatively termed Caffey disease, is a disorder characterized by inflammatory cortical hyperostosis that is prevalent in the first 6 months of life and named after Dr. Caffey, one of the pioneers in pediatric radiology [43, 44]. Most cases are attributable to a specific mutation in the type I collagen gene. The clinical features include painful soft tissue swelling over the involved bones. The condition is usually self-limited. The key radiological findings include heavy periosteal hyperostosis affecting the diaphyses of the long bones, ribs, clavicles, and scapulae (Fig. 16). Involvement of the mandible, if present, can help distinguish this disorder from other causes of inflammatory periostitis in infancy.

## Cleidocranial dysplasia

**Fig. 17:**
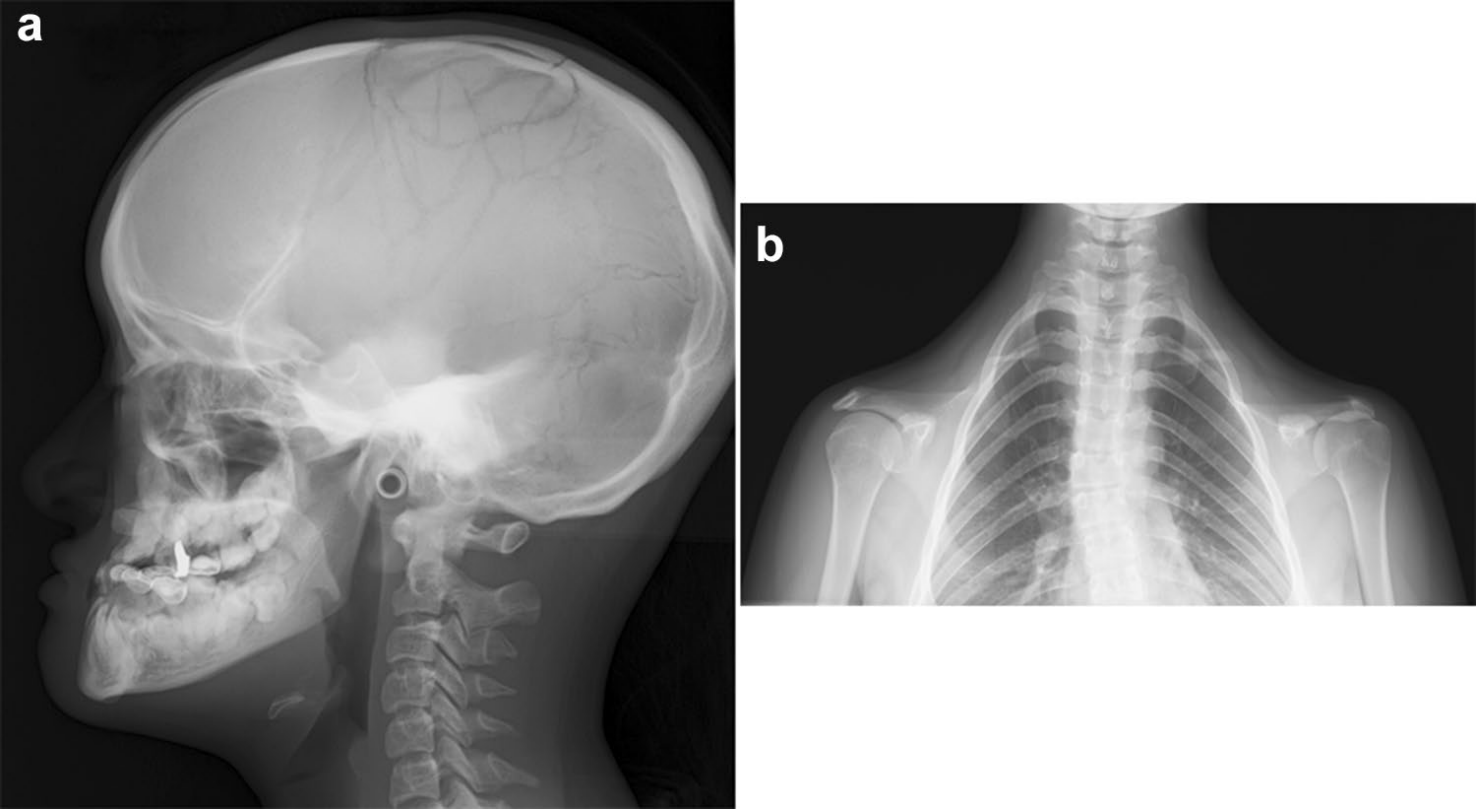
**a**, **b** A 25-year-old female with cleidocranial dysplasia. Radiographs show multiple Wormian bones, absent bilateral clavicles, and a narrow upper thorax

Cleidocranial dysplasia is characterized by clavicular (*cleido-*) defect and late closure of cranial sutures (*cranio-*) [45, 46]. The clinical features include mild craniofacial dysmorphism, delayed closure of the fontanels, dental malformation, and clavicular hypoplasia that causes unusually mobile shoulder (the shoulders can meet in the midline). Otherwise, patients have a benign course without significant physical disabilities. The key radiological findings include defective calvarial ossification with multiple Wormian bones, delayed closure of the fontanels, and delayed closure of the sutures. The aplasia or hypoplasia of the clavicle, most commonly affecting the middle and lateral thirds, can be symmetric or asymmetric (Fig. 17). The scapulae and glenoid are small. Delayed ossification of the pubic bones, ischial bones and sacrum manifests as wide pubic symphyses and sacroiliac joints. Pseudoepiphyses are seen at the metacarpal and metatarsal bases, and the distal phalanges are often hypoplastic.

## Nail–patella syndrome

**Fig. 18:**
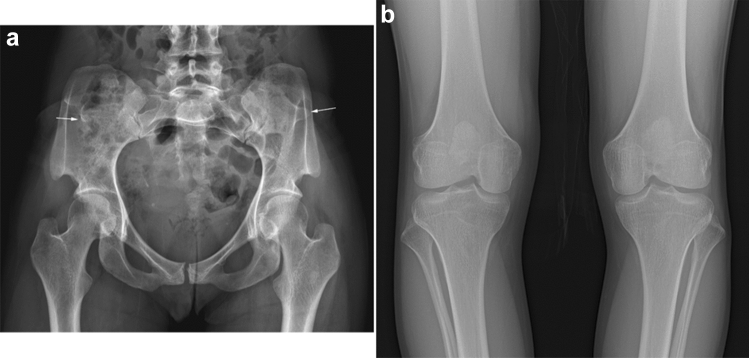
**a**, **b** A 28-year-old female with Nail–Patella syndrome. There are pathognomonic iliac horns (arrows) and hypoplastic patellae

Nail–Patella syndrome is characterized by nail hypoplasia, patellar hypoplasia, and characteristic “iliac horns”—an osseous protuberance arising from the posterior surface of the ilia [47–49]. The hypoplasia of the fingernails is most severe on the thumb and may be the first clinical presentation. Aplastic or hypoplastic patellae are associated with recurrent dislocation. The elbow joint may also be dysplastic. Affected individuals may develop nephropathy in adulthood (approximately one-third of the cases) and need routine renal function tests. The key radiological findings include pathognomonic iliac horns, variable degrees of patellar hypoplasia, and malalignment of the elbow joints (Fig. 18).

## Hereditary multiple exostoses

**Fig. 19:**
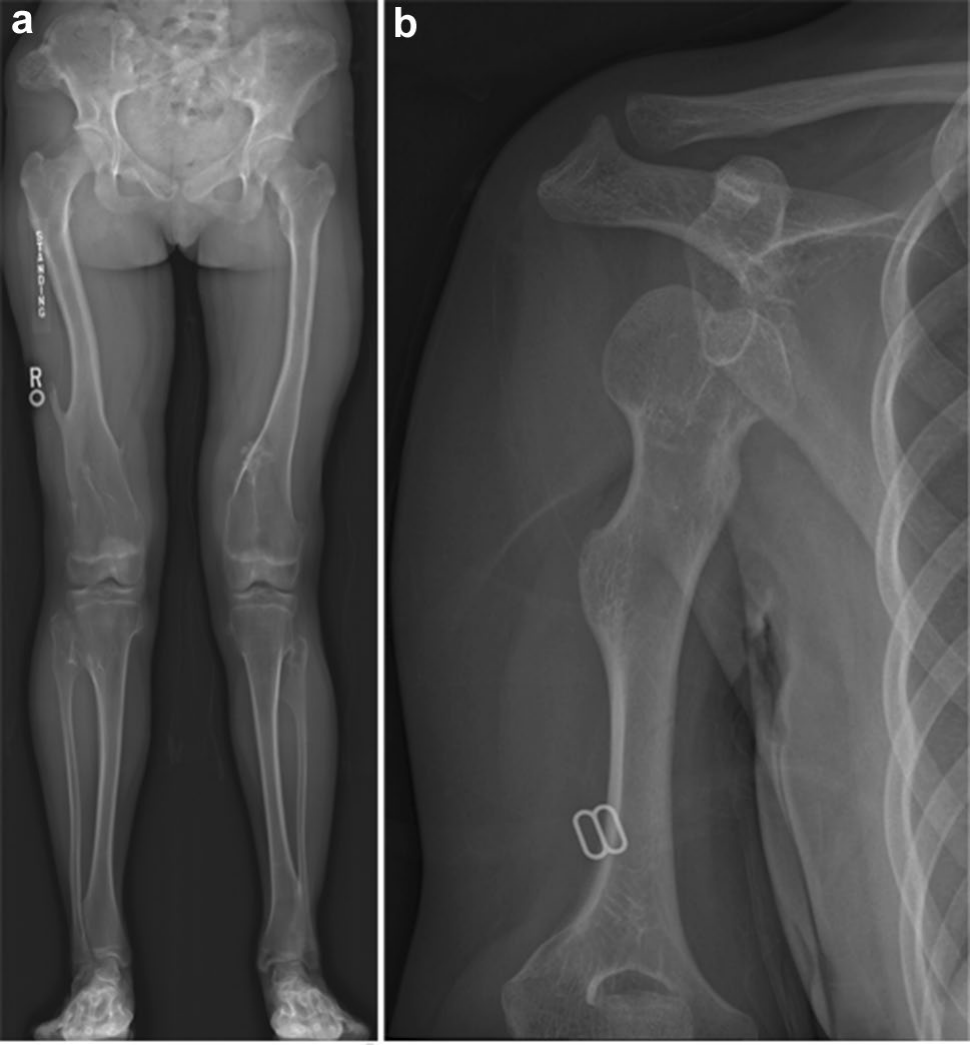
**a** A child with hereditary multiple exostoses. There are multiple exostoses (pedunculated bony protrusions with corticomedullary continuation) of the metaphyses of the long bones. **b** A 20-year-old female with hereditary multiple exostoses. There are sessile exostoses with corticomedullary continuation of the right humerus which impaired the growth of the proximal humeral growth plate

Hereditary multiple exostoses is characterized by multiple osteochondromas or exostoses–osseous protuberances seen at the ends of tubular bones and ribs and at the cartilaginous parts of the axial skeleton, such as the scapular wings and iliac crests [50–52]. As with solitary osteochondroma, the bone projection may cause nerve compression, irritation of tendons, and vascular compromise. These complications are more common in hereditary multiple exostoses. Malignant transformation may also occur with a 1–3% incidence of transformation to chondrosarcoma. The key radiological features are sessile or pedunculated osseous protrusions with corticomedullary continuity with the underlying bone (Fig. 19). Osteochondromas involving the long bones originate in the metaphysis and migrate toward the diaphysis; thus, the bone protuberance points away from the joint. Osteochondromas can be associated with failure of tubulation (metaphyseal broadening) and growth disturbance (shortening of affected bones). Cartilage cap evaluation deserves the most consideration in interpretation as a thick cartilage cap > 1.5 cm has a higher chance of malignant transformation; this is best evaluated on MR T2-weighted images with fat saturation. Continued growth after skeletal maturity, rapid increase in size, destruction of the mother bone, a variegated appearance, or presence of new pain are also worrisome for malignant degeneration.

## Dysplasia epiphysealis hemimelica (Trevor disease)

**Fig. 20:**
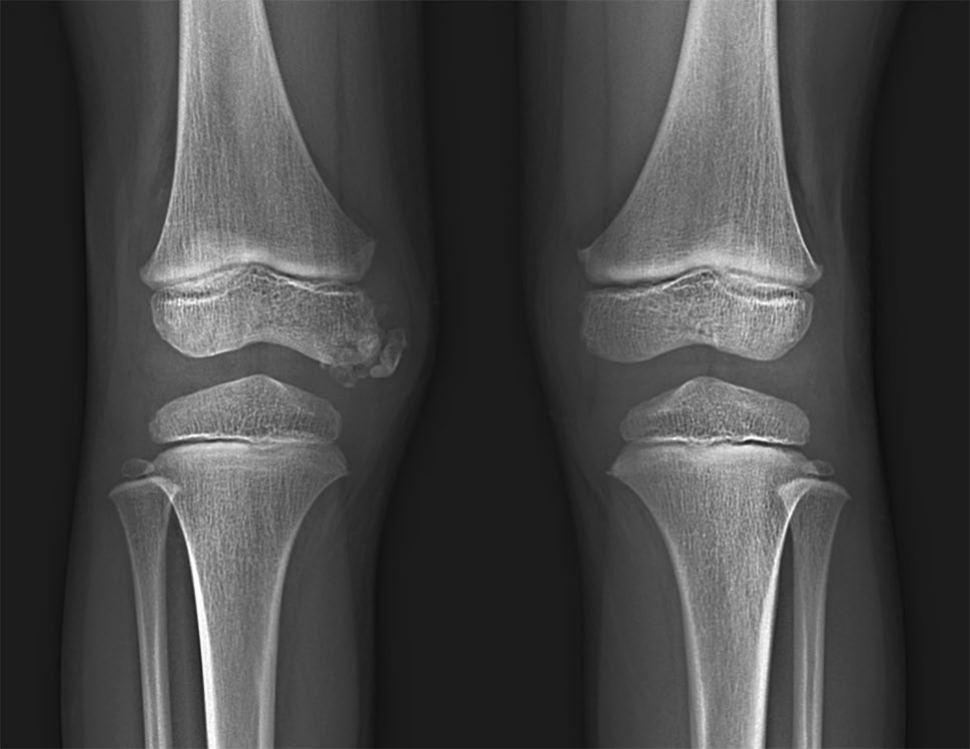
A 5-year-old boy with dysplasia epiphysealis hemimelica (Trevor’s disease). Radiograph shows osseous overgrowth arising from the right medial femoral condyle

Dysplasia epiphysealis hemimelica or Trevor disease is characterized by asymmetric overgrowth of the epiphyseal cartilage [53]. The histologic finding is the same as that of osteochondroma. The clinical features include a painless osseous mass, most commonly around the knee or ankle joints, associated with restricted motion and deformity of the affected joint. The radiological finding is a focus of osseous overgrowth arising from one side of the affected epiphysis, more commonly involving the medial rather than lateral aspect of the involved bone (Fig. 20). Multiple ossification centers can be present. These findings later result in a larger-than-normal epiphysis.

## Enchondromatosis (Ollier disease and Maffucci syndrome)

**Fig. 21:**
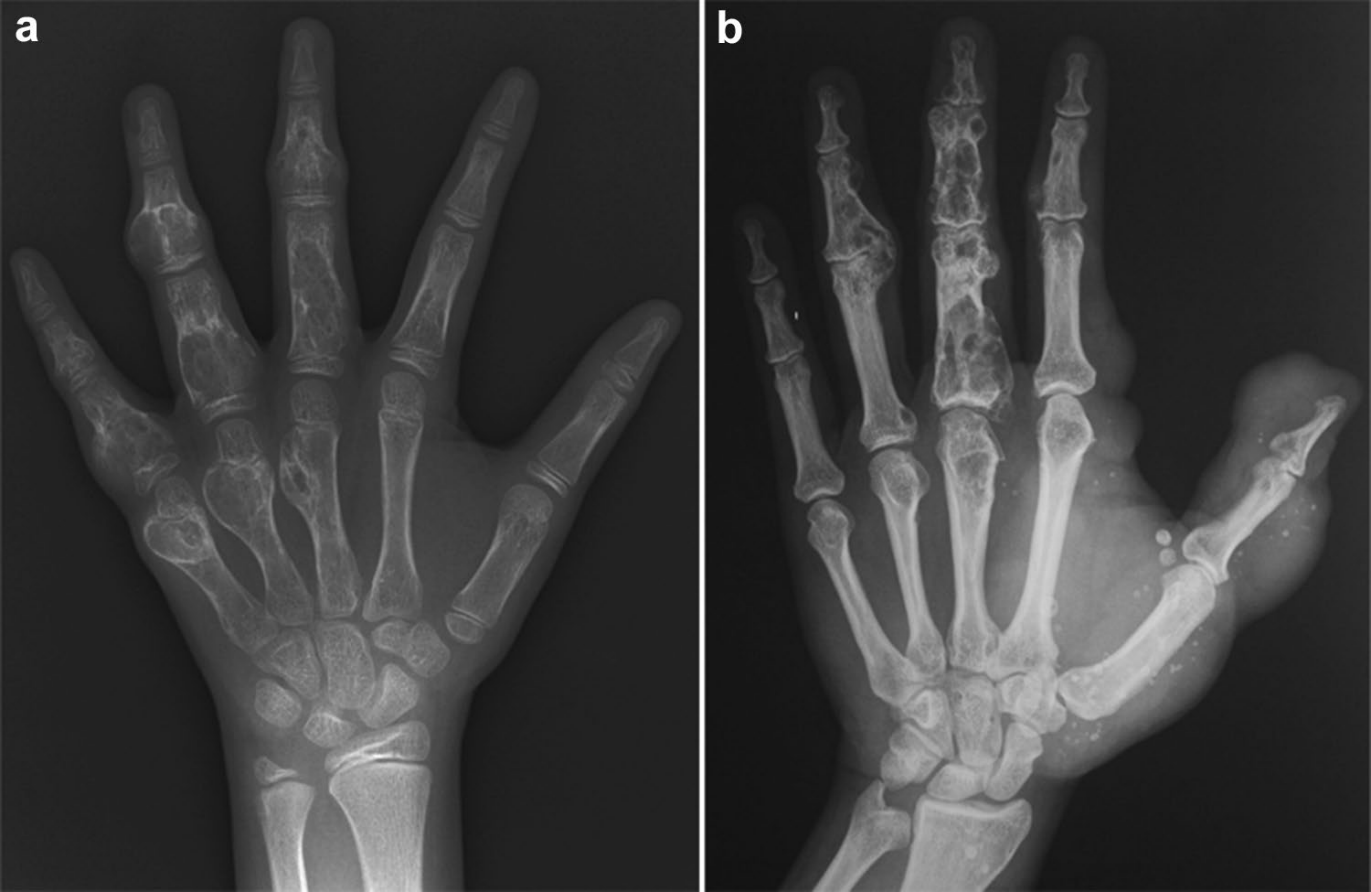
**a** An 8-year-old child with enchondromatosis (Ollier disease). Radiograph shows multiple expansile metaphyseal lucent lesions in the third to fifth phalanges. **b** A young adult with Maffucci syndrome. Radiograph shows multiple enchondromatosis in the second to fourth digits with soft tissue masses associated with multiple foci of round phleboliths compatible with hemangiomas

Enchondromatosis, alternatively termed Ollier disease, is characterized by multiple enchondromas—lesions containing chondroid matrix predominantly in the metaphyses, typically in a unilateral or asymmetric distribution [54]. The clinical feature is tumefaction of the extremities, most commonly of hands and/or feet, occasionally associated with pathologic fractures. Enchondromas of the short tubular bones present as oval geographic radiolucent lesions in the central meta-diaphyses, which may contain “arcs and rings” calcification indicating the presence of a chondroid matrix (Fig. 21a). Enchondromas of the long tubular bones are depicted as columns of linear lucency extending from the growth plate to the metaphysis. MRI shows well-defined lobulated areas of hyperintensity on T2-weighted imaging, as with other cartilaginous lesions. As with osteochondroma, enchondroma may impair the growth plate and cause limb asymmetry. The combination of enchondromatosis and multiple hemangiomas is called Maffucci syndrome (Fig. 21b) [55]. Both Ollier disease and Maffucci syndrome are associated with a higher incidence of malignant transformation to chondrosarcoma. As a rule, no new enchondroma appears after puberty; thus, malignant transformation should be suspected when “new growth” of an enchondroma is seen in adulthood.

## Polyostotic fibrous dysplasia (McCune–Albright syndrome, Mazabraud syndrome)

**Fig. 22:**
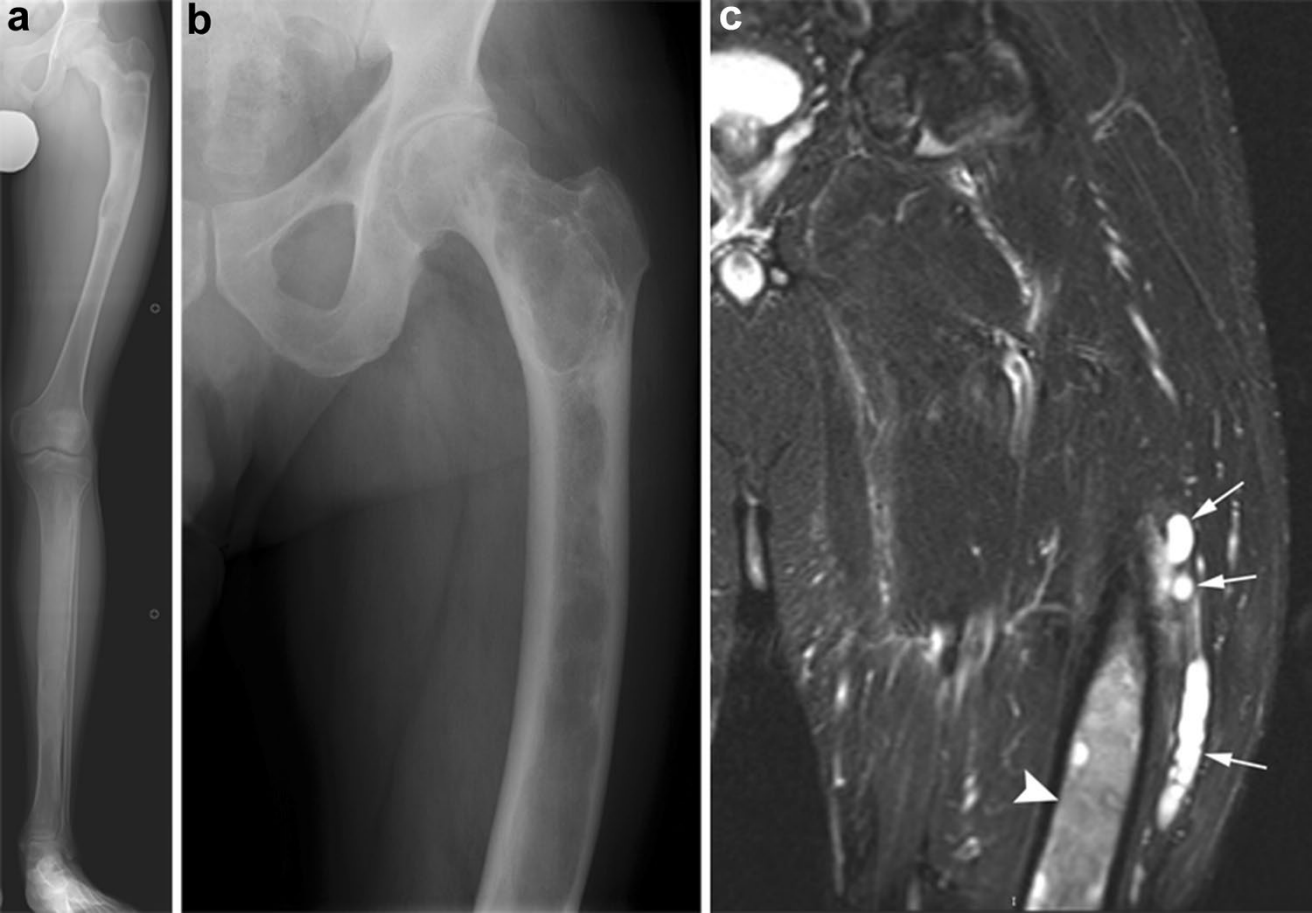
**a** A 13-year-old boy with polyostotic fibrous dysplasia accompanied by café-au-lait spots. Radiograph shows elongated and expansile “ground glass” lesions replacing the bone marrow in the proximal femoral diaphysis and the tibial diaphysis. **b**, **c** A 59-year-old male with Mazabraud syndrome. Radiograph shows fibrous dysplasia involving the femur with coxa varus angulation, giving what is called a “shepherd crook deformity”. Coronal STIR image showing intramuscular hyperintensity compatible with intramuscular myxomas (arrows) as well as heterogeneous hyperintense signal in the femur compatible with fibrous dysplasia (arrowhead)

McCune–Albright syndrome is characterized by a triad of café-au-lait spots, premature puberty, and polyostotic fibrous dysplasia [56–58]. Fibrous dysplasia of the extremities may be associated with pain, pathologic fractures, and deformity of affected bones. Craniofacial involvement may cause proptosis, facial asymmetry, blindness, and deafness. The radiological finding characteristic of fibrous dysplasia is a geographic, elongated, and expansile “ground glass” lesion replacing the bone marrow (Fig. 22a). No periostitis should be present. A proximal femoral lesion with bowing is often described as “shepherd’s crook deformity”. CT may better demonstrate craniofacial lesions that can be mixed sclerotic and lytic in appearance. Mazabraud syndrome is characterized by fibrous dysplasia associated with single or multiple intramuscular myxomas in the vicinity of the bone lesions (Fig. 22b, c). Intramuscular myxomas show intramuscular hyperintensity on T2-weighted imaging [59, 60].

## Conclusions

We have reviewed classic radiological findings and patterns of common skeletal dysplasias. The Aunt Minnie, or pattern recognition approach based on radiographs, is the key toward the correct diagnosis. CT and MRI may provide additional information in selected conditions. Awareness of these distinctive radiological patterns not only helps one excel on the board exam, but also helps you with your patients throughout your career.
